# Metabostemness: Metaboloepigenetic reprogramming of cancer stem-cell functions

**DOI:** 10.18632/oncoscience.113

**Published:** 2014-12-26

**Authors:** Javier A. Menendez, Bruna Corominas-Faja, Elisabet Cuyàs, Tomás Alarcón

**Affiliations:** ^1^ Metabolism & Cancer Group, Translational Research Laboratory, Catalan Institute of Oncology (ICO), Girona, Catalonia, Spain; ^2^ Girona Biomedical Research Institute (IDIBGI), Girona, Catalonia, Spain; ^3^ Computational & Mathematical Biology Research Group, Centre de Recerca Matemàtica (CRM), Barcelona, Catalonia, Spain; ^4^ Departament de Matemàtiques, Universitat Autònoma de Barcelona (UAB), Barcelona, Catalonia, Spain

**Keywords:** Oncometabolites, reprogramming, stemness, cancer, stem cells, cancer stem cells

## Abstract

Cancer researchers are currently embarking on one of their field's biggest challenges, namely the understanding of how cellular metabolism or certain classes of elite metabolites (e.g., oncometabolites) can directly influence chromatin structure and the functioning of epi-transcriptional circuits to causally drive tumour formation. We here propose that refining the inherent cell attractor nature of nuclear reprogramming phenomena by adding the under-appreciated capacity of metabolism to naturally reshape the Waddingtonian landscape's topography provides a new integrative metabolo-epigenetic model of the cancer stem cell (CSC) theory.

By emphatically underscoring the similarities in nuclear reprogramming pathways involved in the generation of induced pluripotent stem cells (iPSCs) and cancer stem cells (CSCs), one of the conclusions drawn from the 2013 Nature-Ludwig Institute for Cancer Research Conference on “*Nuclear Reprogramming and the Cancer Genome*” has been to suggest a new cancer hallmark, namely mutations or expression changes in metabolic genes that are implicated in the regulation of DNA methylation plasticity [1].

## Oncometabolites and the stem cell origin of cancers: A Darwinian view

In cancers with a stem cell origin such as haematopoietic malignancies, gain-of-function isocitrate dehydrogenases (IDH) mutations generating the oncometabolite 2-hydroxyglutarate (2HG) lead to global hypermethylation [2]. This activity prevents the demethylation of genes that are implicated in differentiation, consequently promoting a stabilization of undifferentiated and self-renewing cellular states that may be targetable and expanded by later transforming mutations. For most solid tumours with non-stem cell origins, including liver, breast, lung, pancreatic and prostate cancer, in which mutations in metabolic genes are not widespread, it was alternatively proposed that an intact metabolic function of IDH would be necessary to maintain DNA methylation plasticity and a flexible epigenetic landscape [1].

The latter proposal, however, can be somewhat difficult to reconcile with the actual functioning of the oncometabolite 2HG in solid tumours such as intrahepatic cholangiocarcinomas (IHCCs) [3] and breast carcinomas (BCs) [4]. IHCC is a deadly liver malignancy in which highly prevalent 2HG-producing IDH mutations subvert the hepatocyte differentiation/quiescence program to create a persistent pre-neoplastic state, which is primed for transformation into adenocarcinoma by additional oncogenic mutations [3]. In the absence of IDH mutations, the accumulation of 2HG is part of the *c-Myc*-driven metabolic reprogramming observed in biologically aggressive BCs that exhibit globally increased DNA methylation [4]. The hypermethylation phenotype of 2HG-overexpressing IHCC and BC is characterised by a strong enrichment of a stem cell-like transcriptional signature [3, 4]. Why do oncometabolic traits convergently “encode” an immature, stem-like program in cancer tissue, regardless of the stem cell/non-stem cell source?

It has been argued that the cell-of-origin dictates the metaboloepigenetic relationship between nuclear reprogramming and the generation of CSCs [1], an *ad hoc* evolutionary assumption in which natural forces might select for either “hierarchic” or “dynamic” epigenetic landscapes depending on their stem cell or non-stem cell origin, respectively. Instead, we here propose that refining the inherent cell attractor nature of nuclear reprogramming phenomena [5-8] by adding the under-appreciated capacity of metabolism to naturally reshape the Waddingtonian landscape's topography provides a new integrative perspective of the CSC theory that is free of any *ad hoc* argumentation.

## Metaboloepigenetic reprogramming of cancer stem cells: A Waddingtonian view

**Figure 1:**
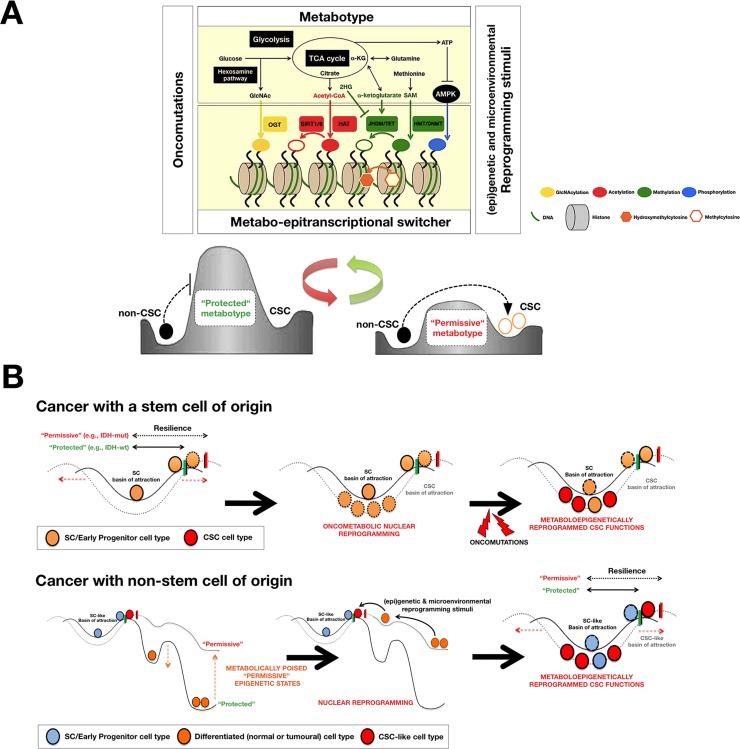
A Waddingtonian perspective of the metabolo-epigenetic reprogramming of stemness in cancer tissues A. A highly active crosstalk process between certain metabotypic features, elite metabolites (e.g., oncometabolites), and epigenetics could allow the causal integration of the metabolism with genetic programs to generate CSC functions via pathological nuclear reprogramming [1, 9]. The acquisition of stemness in cancer tissues might not only be hard-wired by the mutational landscape but also by the pivotal regulatory role of the cellular metabotype. This relationship, in turn, can remove, diminish, or modify the nature of the molecular barriers present in Waddington's epigenetic landscapes, thus allowing cells to more easily (re-)enter into CSC cellular macrostates. In this metabostemness framework, even modest changes in the “protected” *versus* “permissive” nature of the cellular metabotype are expected to produce a considerable change in the global kinetic efficiency of the CSC reprogramming process. The cellular metabotype can be described by means of variability criteria, such as the presence or absence of particular metabolites (e.g., oncometabolites such as 2HG), the concentration levels of certain metabolites, the relative levels or ratios between specific metabolites, metabolic profiles or even spatio-temporal flux distributions of metabolites (e.g., N-acetylglucosamine [GlcNAc] for histone GlcNAcylation, the NAD^+^/NADH ratio for sirtuin histone deacetylase activities, acetyl-CoA as a donor for histone acetylation, alpha-ketoglutarate as a cofactor for histone and DNA demethylation reactions, S-adenosylmethionine [SAM] as a donor for DNA methylation, or ATP/AMP-regulated chromatin translocation of AMPK for histone phosphorylation). B. The cellular metabotype may act as “starter dough” that renders any type of cell-of-origin susceptible to the epigenetic rewiring required for the acquisition of refractoriness to differentiation [9]. This modification can significantly alter the efficiency and speed of CSC reprogramming in cancers with (top) and without (bottom) a stem cell origin by lowering the barriers of the epigenetic landscape and increasing the size of the basins of attraction, which are necessarily located in the developmentally immature, stem-like regions of the “higher mountains” of the landscape. From a therapeutic perspective, small perturbations in a particular metabolic pathway or metabolite might have drastic consequences on the formation, maintenance, and evolution of CSC cellular states. Indeed, the unexpected applications for biguanides in oncology might closely relate to their metabolic effects during the induction of CSCs [15-17]. (OGT: O-linked N-acetylglucosamine transferase; SIRT1/6: NAD^+^-dependent SIRTUIN histone deacetylases (HDACs); HAT: Histone acetyltransferases; JHDM/TET: Jumonji-C domain containing histone demethylases (HDMs)/Ten-eleven translocation (Tet) methylcytosine dioxygenases; HMT/DNMT: Histone methyltransferase/DNA methyltransferases)

We recently coined the term “metabostemness” to refer to the metabolic parameters causally controlling or functionally substituting the epi-transcriptional orchestration of CSC nuclear reprogramming [9]. A central metabostemness element is the metabolo-epitranscriptional switcher, which decodes the metabolism's ability to interfere with the developmental Waddington's “buffering” and “canalization” *via* the removal, lowering or modification of landscape “energy” barriers (Fig. 1A). Two key operational features determine the functioning of metabostemness: a.) Two primary epigenetic codes, DNA methylation and histone modification, and the consequent epigenetic regulation of cell differentiation genes are the pivotal molecular events that account for the regulatory effects of metabolism on nuclear reprogramming [10], and b.) the switcher is responsive not only to *bona fide* oncometabolites but also to more common primary metabolites employed by chromatin-remodelling enzymes [9, 10], implying that even small changes in the landscape's fine topography (e.g., metabolically driven leaning of a slope in a separating barrier) could sufficiently place cells into the basins of CSC attractors without the intervention of mutational events. In cancers with a stem cell origin, certain metabotypes might provoke cells to get stuck very near or in the same state-space of attractors of previously normal stem cells (e.g., by increasing the size of the basins and, therefore, the resilience of stem-like states), which will subsequently increase the probability of undifferentiated cells targetable by pro-proliferative oncomutations (Fig. 1B, *top*). For cancers with non-stem cell origins, certain metabotypes can permissively alleviate the “uphill”, unfavourable developmental process of “jumping back” from non-CSC differentiated valleys to high-altitude CSC attractors while concomitantly promoting the ground-state character of self-maintaining CSC-like states (Fig. 1B, *bottom*).

Cell types can occupy attractors even after the metabolic stimulus triggering the transition disappears, a “memory effect” in a multi-attractor, flexible landscape that explains why the transient occurrence of CSC-promoting events (e.g., hypoxia- or acidic pH-induced loss of pro-differentiation factors) [11, 12] are sufficient to generate a lasting CSC population (e.g., long-lived dormant stem cell-like cells). If certain metabotypes confer a proliferative advantage on cells trapped in abnormal attractors, somatic evolution can further deepen them and, therefore, promote their over-occupancy by specific cancer cell types (e.g., IDH-mutated IHCC with stem cell features and exhibiting bile duct differentiation) [3].

## CONCLUSIONS

CSC states can be viewed as inherently inevitable epigenetic deviations of Waddington's developmental landscapes in which metabolism modifies the probability that not only stem cells/early progenitors but also normal or tumour-differentiated cells can find either pre-existing or *de novo* occupied, self-organising attractors encoding dynamically robust CSC signatures that somatic evolution alone cannot explain. Oncometabolites can therefore operate as the metabolic basis for the epigenetic landscape of tumour-initiating events regardless of the stem cell/non-stem cell source and without the requirement of mutations in metabolic enzymes [13, 14].
